# GxEsum: a novel approach to estimate the phenotypic variance explained by genome-wide GxE interaction based on GWAS summary statistics for biobank-scale data

**DOI:** 10.1186/s13059-021-02403-1

**Published:** 2021-06-21

**Authors:** Jisu Shin, Sang Hong Lee

**Affiliations:** 1 Australian Centre for Precision Health, University of South Australia Cancer Research Institute, University of South Australia, Adelaide, SA 5000 Australia; 2 UniSA: Allied Health and Human Performance, University of South Australia, Adelaide, SA 5000 Australia

**Keywords:** GxE interaction, Whole-genome approach, Biobank-scale data, Reaction norm model, LDSC

## Abstract

Genetic variation in response to the environment, that is, genotype-by-environment interaction (GxE), is fundamental in the biology of complex traits and diseases. However, existing methods are computationally demanding and infeasible to handle biobank-scale data. Here, we introduce GxEsum, a method for estimating the phenotypic variance explained by genome-wide GxE based on GWAS summary statistics. Through comprehensive simulations and analysis of UK Biobank with 288,837 individuals, we show that GxEsum can handle a large-scale biobank dataset with controlled type I error rates and unbiased GxE estimates, and its computational efficiency can be hundreds of times higher than existing GxE methods.

**Supplementary Information:**

The online version contains supplementary material available at 10.1186/s13059-021-02403-1.

## Background

The success of the human genome project has led to a paradigm shift in the complex trait analysis that focuses on the genome-wide association studies (GWAS) [1]. GWAS have been incredibly successful at identifying genome-wide significant single nucleotide polymorphisms (SNPs) that are associated with causal variants underlying complex traits [2, 3]. Moreover, whole-genome approaches, using all common SNPs across the genome, have been useful to dissect the genetic architecture of complex traits, e.g., SNP-based heritability and genetic correlation [4]. However, the analytical modeling used in GWAS and whole-genome approaches usually assumes that there is no genotype-by-environment interaction (GxE), which can be often violated against the true genetic architecture of complex traits. Indeed, interaction is fundamental in biology, and there has been increasing interest in estimating GxE, using genome-wide SNPs [5–7].

Current state-of-the-art whole-genome methods for estimating GxE include genotype-covariate interaction genomic restricted maximum likelihood (GREML) and random regression GREML [8]. Recently, a multivariate reaction norm model (RNM) has been introduced [9], which can disentangle GxE from genotype-environmental correlation, providing more reliable GxE estimations. These methods typically employ the GREML approach that requires individual-level genotypes and is computationally intensive. Especially when using biobank-scale data, the approach becomes computationally intractable.

To reduce the computational limitation of GREML, linkage disequilibrium score regression (LDSC) was introduced to estimate SNP-based heritability and genetic correlation [10]. LDSC is computationally efficient and requires no individual-level genotypes. Instead, it uses GWAS summary statistics, regressing the association test statistics of SNPs on their LD score. However, existing LDSC methods are limited to additive models only [11–14].

In this study, we propose a novel approach to estimate the phenotypic variance explained by genome-wide GxE based on GWAS summary statistics (GxEsum) for a large-scale biobank dataset, correctly accounting for genotype-environment correlation and scale effects. In simulated and real data analyses, we show that the computational efficiency of the proposed approach is substantially higher than RNM, an existing GREML-based method, while the estimates are reasonably accurate and precise. Because of this computational advantage, GxEsum may be an efficient tool to estimate GxE that can be applied to large-scale data across multiple complex traits.

## Results

### Method overview

We propose a method to estimate the phenotypic variance explained by the whole-genome GxE, based on GWAS summary statistics, referred to as GxEsum. GxEsum can be a computationally efficient RNM using an extension of the LDSC approach. While the existing LDSC approach is designed to use estimated additive SNP effects in GWAS summary statistics (Additional file 1: Note S1), GxEsum requires summary statistics of SNP-by-environment interaction effects. For SNP effects modulated by an environment, the expected chi-square statistic (${\chi}_j^2$) is:

$$ \mathrm{E}\left[{\chi}_j^2|{\ell}_j\right]=\frac{N{\sigma}_{g_1}^2}{M}\ast {\ell}_j+1+2\left({\sigma}_{g_1}^2+{\sigma}_{\tau_1}^2\right) $$

where N is the number of individuals, M is the number of SNPs, ${\sigma}_{g_1}^2$ is the variance due to GxE, ${\sigma}_{\tau_1}^2$ is the variance due to residual heterogeneity or scale effects caused by residual-by-environment interaction (RxE), and *ℓ*_*j*_ is the LD score at the variant *j* that can be estimated from a reference panel (please see the “Methods” section for a full derivation of this equation). The ${\chi}_j^2$ test statistics correspond to the regression coefficient for the interaction between the *j*th SNP and the environmental covariate (E). The outcome trait is pre-adjusted for confounders and the main effects of E, and then a regression model with the main and interaction effects is run by SNP-by-SNP. If chi-square statistics from GWAS are regressed on LD scores, non-genetic interaction effects (${\sigma}_{\tau_1}^2$) are captured by the intercept, from which GxE (${\sigma}_{g_1}^2$) can be disentangled. Consequently, GxE effects estimated by GxEsum are equivalent to that adjusted for RxE when using RNM [9].

To validate the proposed model, i.e., GxEsum, we used various simulations based on real genotype data (see Additional file 1: Note S2 for a full description of the simulation models). In simulations with and without GxE, we assessed the type l error rates and the accuracy of estimated GxE. We deliberately generated confounding effects such as genotype-environment (G-E) correlation, RxE, and residual-environment (R-E) correlation, to see if the type l error rate and the accuracy of GxEsum were affected by these confounding factors.

In the real data analysis, we used the UK Biobank data with 288,837 unrelated individuals after stringent quality control. Subsets of the data with various sample sizes were analyzed to compare the precision (i.e., power) and the computational efficiency of GxEsum and GREML-based GxE model (i.e., RNM).

Finally, we show how the genetic effects of a complex trait (e.g., BMI, hypertension, or type 2 diabetes) are modulated by environment (e.g., neuroticism score, alcohol intake frequency, physical activity, or age) by using the proposed method.

### Simulations

For a continuous trait, under the null (no GxE), whether or not there were confounding effects (RxE and G-E and R-E correlations), the type I error rate of GxEsum was not significantly inflated (Table 1). Note that the use of 500 replicates for each simulation scenario can detect a type I error of greater than 0.07 or less than 0.03 as significantly different from 0.05, using the binomial distribution theory [15, 16]. Even with larger confounding effects (Additional file 1: Table S1), there was no inflation for the type I error rate of GxEsum.

**Table 1:** Type I error rates of GxEsum to detect GxE at a significance threshold of p-value < 0.05

Scenarios	Type l error rate
Var(GxE) = 0, var(RxE) = 0	0.066
Var(GxE) = 0, var(RxE) = 0, G-E correlation = 0.1	0.064
Var(GxE) = 0, var(RxE) = 0, R-E correlation = 0.1	0.044
Var(GxE) = 0, var(RxE) = 0, G-E correlation = 0.1, R-E correlation = 0.1	0.056
Var(GxE) = 0, var(RxE) = 0.1	0.044
Var(GxE) = 0, var(RxE) = 0.1, G-E correlation = 0.1	0.034
Var(GxE) = 0, var(RxE) = 0.1, R-E correlation = 0.1	0.028
Var(GxE) = 0, var(RxE) = 0.1, G-E correlation = 0.1, R-E correlation = 0.1	0.054
Average	**0.049**

We simulated phenotypic data based on a real genotypic dataset (ARIC GWAS) including 7263 participants with 583,085 SNPs, using various scenarios. The phenotypes were standardized such that the phenotypic mean was 0 and the phenotypic variance was 1. Type I error rate (i.e., false-positive) was estimated from 500 replicates for each scenario

*GxE* genotype-by-environment interaction, *RxE* residual-by-environment interaction, *G-E correlation* genotype-environment correlation, *R-E correlation* residual-environment correlation

In the simulation with non-zero interactions, estimated GxE (g1) was not remarkably different from the true values whether there were significant G-E and R-E correlations or not (see Additional file 1: Figure S1). It was noted that the RxE component was correctly captured by the intercept and not confounded with GxE estimates even when using non-normal environmental variables (Additional file 1: Note S3 and Tables S2 and S3). In the absence of RxE, estimated GxE was also unbiased (Additional file 1: Figure S2). The estimated GxE seemed robust to different values of G-E and R-E correlations ranging from 0.05 to 0.2, respectively (Additional file 1: Figures S3 and S4).

On the other hand, the estimated main genetic variance (g0) was slightly biased especially when using a large G-E or R-E correlation (Additional file 1: Figure S4). This is probably because of the fact that the main genetic effects are over-adjusted for the environment due to the large correlations (between the trait and environment) in the model.

We also validated that there was no inflation for the type I error rate when applying GxEsum to binary (disease) traits (Table 2 and Additional file 1: Table S4), showing that GxEsum appears to be robust to false positives in the scenarios of various confounders. In addition, we estimated the variance component of GxE on the observed scale and transformed it to that on the liability scale, using Robertson transformation [17]. As shown in Additional file 1: Figure S5, the transformed estimates were close to the true simulated values on the liability scale, although the precision of estimates (represented as 95% CI) was shown to be decreased when the population prevalence approached an extreme (e.g., k = 0.025). GxE estimates were biased when simulating a large effect size of GxE (e.g., 10% of phenotypic variance explained by GxE) in the case of k = 0.025 (Additional file 1: Figure S6) although they were mostly unbiased in the case of k = 0.1 (Additional file 1: Figure S7). The level of biasedness appeared to be increased when there were RxE effects (Additional file 1: Figure S6). Finally, caution should be given in interpreting GxE estimates when there are large confounding effects such as substantial G-E and R-E correlations (Additional file 1: Figure S8). The inflated GxE estimates were probably due to the fact that the phenotypes were over-adjusted for the environment in the model because of the correlation between the main trait and environment (G-E and R-E correlations). This resulted in a reduced phenotypic variance (Additional file 1: Figure S9), hence inflated GxE estimates.

**Table 2:** Type I error rates of GxEsum when using binary disease traits with various population prevalence

Scenarios	Population prevalence (k)	Type I error rate
Var(GxE) = 0, Var(RxE) = 0	0.025	0.052
	0.05	0.042
	0.1	0.076
	0.5	0.054
Var(GxE) = 0, Var(RxE) = 0.1 (on the liability scale)	0.025	0.044
	0.05	0.036
	0.1	0.050
	0.5	0.052
Average		**0.050**

We simulated quantitative phenotypic data based on a real genotypic dataset (ARIC GWAS) including 7263 individuals with 583,085 SNPs. The phenotypes were standardised such that the mean was 0 and variance was 1, for which we applied the liability threshold model to generate affected or unaffected disease status for each individual, using various values for the population prevalence (k = 0.025, 0.05, 0.1 or 0.5). Type I error rate at a significance threshold of *p*-value < 0.05 was estimated from 500 replicates for each scenario and population prevalence

Nevertheless, those confounders including RxE interaction and G-E/R-E correlations would not produce false positives whether using continuous quantitative or binary responses as shown above (also see Additional file 1: Tables S1 – S4). We additionally tested if the type I error rate of GxEsum was controlled when there is a collider bias, which is a concern especially when using a self-report study (e.g., UK Biobank data) [18, 19]. In simulations with collider bias, although estimated SNP heritability was substantially (and unrealistically) underestimated (Additional file 1: Figures S10 and S11), the type I error rate of GxEsum was well controlled whether using continuous or binary responses (Additional file 1: Tables S5 and S6).

The estimated variance of the main genetic effects was mostly unbiased when using binary disease traits without G-E/R-E correlations (Additional file 1: Figure S12). When there were significant G-E and/or R-E correlations, the estimated variance of the main genetic effects appeared to be underestimated especially when there was RxE interaction (Additional file 1: Figure S13), which confirmed the fact that the main genetic effects are over-adjusted for the environment due to the correlations between the trait and environment (Additional file 1: Figure S9).

### Precision and computational efficiency

GxEsum uses the Wald test to get a p-value for the null hypothesis, i.e., the absence of GxE interaction, using an estimated GxE variance and its standard error. Therefore, the power of the method is closely related to the precision.

**Fig. 1:**
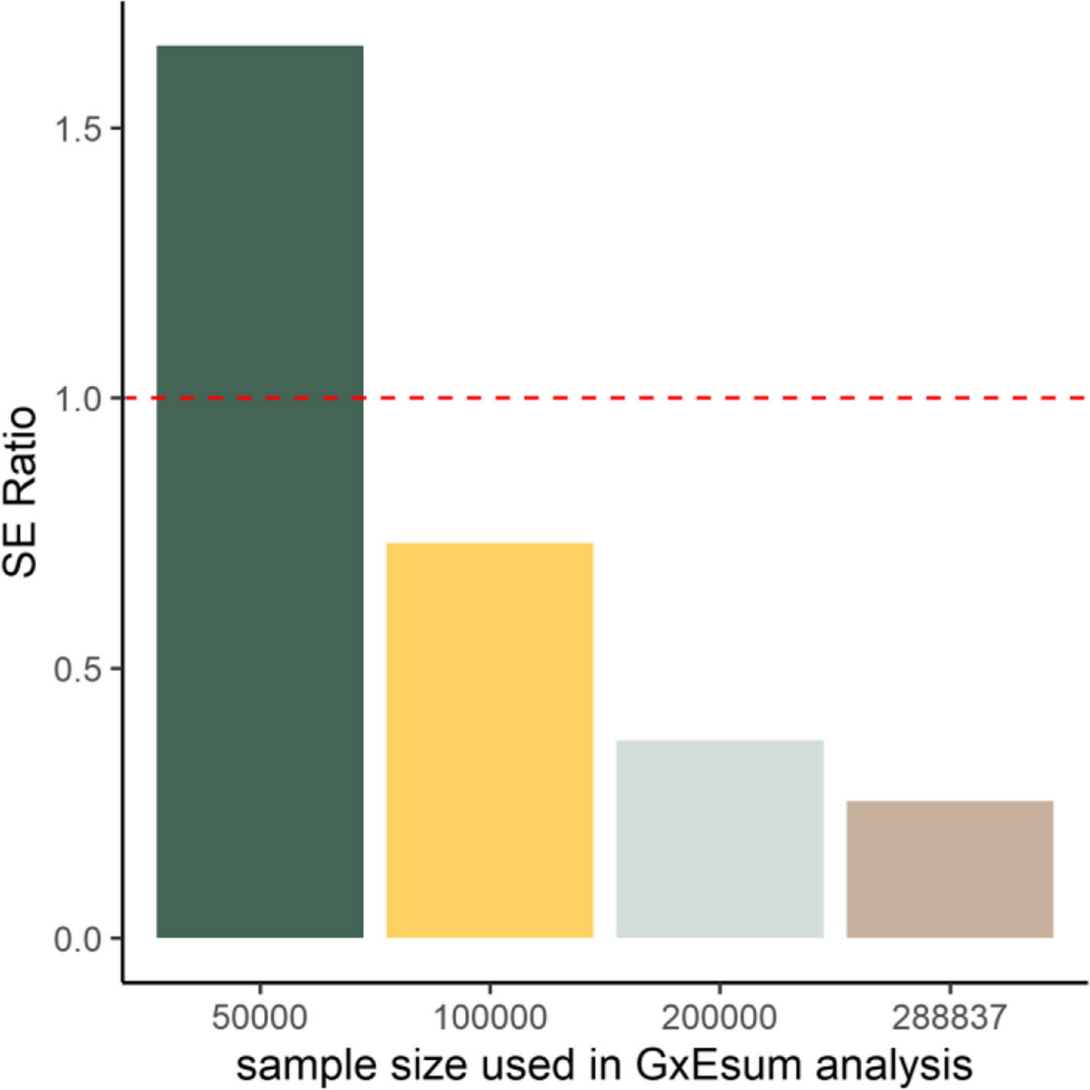
The ratio of standard error (SE) from GxEsum to that from RNM using UK Biobank data. The SEs of GxE variance estimated from GxEsum with various sample sizes ranging from 50,000 to 288,837 were obtained, and they were compared to the SE of GxE variance estimated from RNM with a sample size of 50,000. The dashed horizontal line represents the ratio as 1

The precision was assessed by comparing the standard error (SE) of GxEsum and RNM estimates. The SE of GxEsum was obtained from the LDSC software (using a jackknife method). The SE of RNM for the GxE component can be obtained from the information matrix [20] or from a well-established theory [21] (see Additional file 1: Table S7). Figure 1 shows that the SE of GxEsum was 1.65 times higher than that of RNM when using the same sample size of 50,000. However, when the sample size increased for GxEsum up to 288,837, for which RNM estimation is infeasible, the ratio reduced to 0.2. GxEsum can use a larger sample size (e.g., > 1,000,000), for which the ratio is expected to be further decreased, although the largest sample size tested in this study was 288,837 (Additional file 1: Figure S14).

**Fig. 2:**
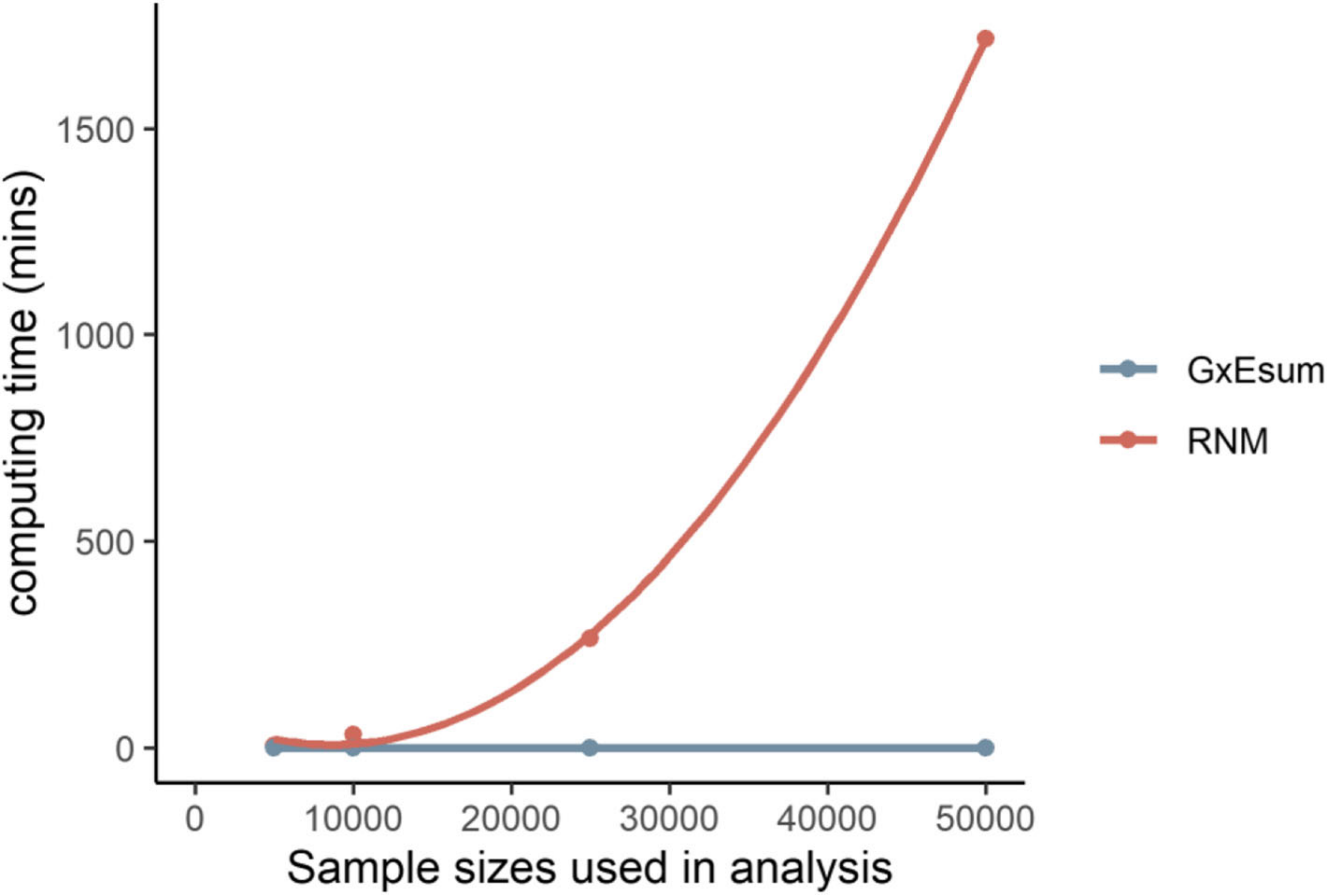
Computing time with various sample sizes used in GxEsum and RNM analyses. As the sample size increases, the computing time of RNM (red) increases exponentially, while that of GxEsum (blue) is almost invariant (less than a minute)

While the precision of GxEsum is competitive with that of RNM, the computational efficiency is dramatically different between the two methods (Fig. 2 and Additional file 1: Table S8). For example, when using a sample size of 50,000, the computing time for RNM was taken more than a thousand times than GxEsum. Even for GxEsum with a sample size of 288,837, its computational efficiency was still substantially higher than RNM with a sample size of 50,000 (Additional file 1: Figure S14 and Table S7). This justifies that GxEsum is a computationally efficient tool that can be applied to biobank-scale data for multiple complex traits and diseases. It is noted that we assumed that preliminary analyses for each method were already done (e.g., GRM for RNM, and LD scores and GWAS for GxEsum) (Additional file 1: Table S8).

### Real data analyses

We applied GxEsum to estimate the genetic effects of body mass index (BMI) that were modulated by an environment such as age, alcohol intake frequency, neuroticism scores, or physical activity. The significant GxE was observed from the analyses using neuroticism scores. On the other hand, we did not find any significant GxE when using age, alcohol intake frequency, and physical activity after Bonferroni correction (Bonferroni p-value = 0.05/10 = 0.005 since there were 10 significance tests in this study) (Table 3). The GxEsum approach applied to a binary disease was conducted using hypertension or type 2 diabetes as the main trait, and BMI, waist-hip ratio (WHR), body fat percentage (BFP), or systolic/diastolic blood pressures (BP) as an environmental variable. Table 3 shows that the genetic effects of hypertension and type 2 diabetes were significantly modulated by BMI, but not by other environmental variables. For a comparison, LDSC estimates (i.e., from the null model without GxE) are also shown in Additional file 1: Table S9.

**Table 3:** Estimates obtained from GxEsum analysis using real data

Main trait	Environmental variable	Main additive genetic variance (${\sigma}_{g_0}^2$)	GxE interaction variance (${\sigma}_{g_1}^2$)	*p*-value for GxE
BMI	Age	0.216 (0.007)	0.004 (0.002)	1.86E−02
	NEU	0.216 (0.007)	0.007 (0.002)	1.61E−05
	PA	0.218 (0.007)	0.003 (0.001)	2.57E−02
	ALC	0.216 (0.007)	0.003 (0.002)	5.98E−02
Hypertension	BMI	0.152 (0.008)	0.006 (0.002)	2.09E−03
	WHR	0.154 (0.008)	0.005 (0.002)	3.21E−02
	BFP	0.151 (0.008)	0.008 (0.003)	2.66E−02
Type 2 diabetes	BMI	0.141 (0.014)	0.085 (0.022)	1.58E−04
	Diastolic BP	0.198 (0.014)	− 0.004 (0.006)	5.38E−01
	Systolic BP	0.204 (0.014)	− 0.006 (0.006)	3.17E−01

We used a quantitative trait (BMI) and binary disease traits (hypertension and type 2 diabetes) because BMI is known to be modulated by age/lifestyle such as NEU, ALC, and PA [8, 22, 23], and hypertension and type 2 diabetes are known to be associated with obese traits such as BMI, WHR, and BP [24, 25]. The p-value is from a Wald test for the estimated GxE variance not being different from zero. The estimates on the observed scale for the binary traits were transformed to those on the liability scale using Robertson transformation [17, 26]. All estimates were from the GxEsum model

*NEU* neuroticism score, *PA* physical activity, *ALC* alcohol intake frequency, *WHR* waist-hip ratio, *BFP* body fat percentage, *BP* blood pressure

Because not all variables were without missing, we imputed missing phenotypes using the mean value for each variable in the analyses, in order to maximize the sample size. In this real data analysis, there was no remarkable difference in the results whether using phenotypic imputation or not although some variables improved their significance, e.g., NEU (Additional file 1: Tables S10 -S12). It is noted that our main phenotypes had a small proportion of missing values, i.e., 0.3%, 7.9%, and 0.2% for BMI, hypertension, and type 2 diabetes, respectively. If the missing rate is substantially high, we recommend excluding the missing values from the analysis, or a better phenotypic imputation method [27] should be used.

## Discussion

In this study, we propose GxEsum, a novel whole-genome GxE method, of which the computational efficiency is a thousand times higher than the existing methods. The estimation of GxE using GWAS summary statistics has great flexibility in the application of the method to multiple complex traits and diseases. The proposed method and theory have been explicitly verified using comprehensive simulations that were carried out for both quantitative trait and binary disease. Moreover, we showed that the type I error rate of the proposed method was not inflated by moderate to severe collider bias [18] that caused a substantial underestimation of heritability shown in our simulation (Additional file 1: Figures S10 and S11).

In the real data analysis, we show that the genetic effects of BMI were significantly modulated by NEU, which agrees with previous studies [9]. It is noted that the significance of GxE was improved because we used a larger sample size, compared with the previous studies. Our result agrees with Robinson et al. [8] who found no significant GxE evidence for age when analyzing BMI using the UK Biobank in which the participants aged 40–69 at the recruitment. However, a dataset with a wider range of ages is desirable, which would increase the power to detect GxE on age. For example, a significant GxE was found in a BMI-age analysis using a dataset including samples aged 18–80 at the recruitment [8].

For hypertension and type 2 diabetes, their causal relationship with BMI has been reported by a number of studies using Mendelian randomization [24, 28, 29]. However, it was not clear if the causal relationship was due to GxE or something else, e.g., unknown non-genetic effects of the disease modulated by BMI status. Here, we show that the causal relationship between hypertension and BMI, and that between type 2 diabetes and BMI reported in the previous studies [24, 28, 29] may be partly due to genome-wide GxE interaction effects. The GxE interaction variance for type 2 diabetes is substantially larger than that for hypertension when using BMI as environmental exposure. This observation agrees with Hyppönen et al. [24] reporting that BMI genetic risk score is more strongly associated with type 2 diabetes than hypertension. In contrast, there is no significant evidence of genome-wide GxE for hypertension-WHR, hypertension-BFP, type 2 diabetes-DBP, or type 2 diabetes-SBP causal relationship that was observed in Mendelian randomization studies [29–31]. This is not totally unexpected because type 2 diabetes or hypertension was reported to be more significantly associated with BMI than other variables [24, 28, 29].

The estimated intercept from GxEsum should be interpreted with caution. We show that estimated intercepts were unbiased from the theoretically predicted values when using the simulation of quantitative traits, as a proof of concept, i.e., the phenotypic variance explained by RxE effects (${h}_{\tau_1}^2$) can be obtained as ${h}_{\tau_1}^2$ = (*intercept* − 1 − 2${h}_{g_1}^2$)/2 from Eq. (4), or more generally, ${h}_{\tau_1}^2$ = (*intercept* −1 − (*k*_*urtosis*_ − 1)${h}_{g_1}^2$)/(*k*_*urtosis*_ − 1) from Eq. (5). However, in real data analyses, there may be additional confounding effects such as scale effects, residual heteroscedasticity, or/and sample heterogeneity that are often attributed to unknown factors. Moreover, when using binary traits, substantial scale effects can be generated (statistical RxE effects) because only the affected and unaffected status are observed and individual differences within the affected or unaffected group are ignored. These additional confounding effects and statistical scale effects are captured and estimated as an intercept in GxEsum [10], resulting in unreliable RxE estimates. It is noted that RxE estimation is not the main interest of GxEsum and can be more reliably estimated in RNM that is designed to model both GxE and RxE.

The existing GxE methods require individual-level genotype data which often has a restriction to share, and their computational burden is typically high. Moreover, it is not clear how they perform when the representativeness of the samples is limited, e.g., selection bias due to a collider in the UK Biobank samples. On the contrary, the proposed approach, GxEsum, is computationally efficient and can detect GxE interaction correctly for both quantitative and binary disease traits even when there is moderate to server collider bias. If GWAS summary statistics of the estimated main additive and interaction effects can be made publicly available, a meta-analysis across multiple cohorts can be possible for an ever-large GxE study (like the context of LDSC SNP heritability meta-analysis). There are some issues that the measure of the environmental variable may not be standardized across study cohorts, and the environmental variable maybe even unavailable in some cohorts. However, these issues can be remedied when the information of exposome that is the standardized measure of all exposures for individuals, complemented to the genome, is available.

There is a GxE method that can use GWAS summary statistics, i.e., VarExp, which is recently published. While VarExp benefits computationally from using GWAS summary statistics, it needs to invert the correlation matrix between SNPs, which prevents from using a large number of SNPs [32]. Furthermore, the theoretical frameworks of GxEsum and VarExp are fundamentally different in that the latter does not account for confounding effects such as scale effects, residual heterogeneity, or RxE that can be captured by the estimated intercept of GxEsum. Finally, the performance of VarExp has been verified with a limited magnitude of interaction effects up to 1.5% and 0.25% of the phenotypic variance for quantitative and binary traits, respectively [32].

Like RNM, GxEsum can fit environmental exposures such that the genetic effects of a trait can be modeled as a nonlinear function of a continuous environmental gradient. The potential modifier of the genetic effects is not limited to environmental exposures but can be extended to novel variables from multi-omics data such as gene expression, protein expression, and methylation data [33, 34]. Polygenic risk scores [35, 36] can also be considered as an environmental variable in the model. This novel approach may allow dissecting a latent biological architecture of a complex trait in a future application of GxEsum.

In the analysis of binary disease traits, estimates on the liability scale, transformed from those on the observed scale using Robertson transformation, should be interpreted with caution. Biased estimates on the liability scale are likely due to the violation of the normality assumption that is essentially required for the Robertson transformation, i.e., large interaction effects can cause a non-normal phenotypic distribution. It is also known that if the transformation involves substantial non-additive effects, it can give biased estimates on the liability scale [17, 26, 37]. However, when non-additive effects are small, the transformation can give reasonably accurate estimates on the liability scale, which is also evidenced by our simulations with small interaction effects. As shown in the real data analysis, the magnitude of genome-wide GxE is not large (< 10% of the phenotypic variance), showing that the bias of transformation due to the assumption violation may not be substantial in general. Nevertheless, it is required to develop a better transformation method for large interaction effects in a further study, e.g., when using multiple environmental variables simultaneously, the interaction effects are aggregated and can be substantially large.

There are a number of limitations to our study. First, like RNM, GxEsum does not determine the causal direction between variables, which can be provided from previous studies or other epidemiologic methods, e.g., Mendelian randomization, as prior information. Second, we only modeled the first order of random regression coefficients with a single environmental variable, and there may be significant additional effects when modeling a higher-order interaction or multiple environmental variables. It is possible to extend the GxEsum model to fit additional quadratic and polynomial terms or multiple environmental variables simultaneously. However, assessing the performance of these advanced models is a formidable task, requiring further study. Third, the estimation for the main genetic effects can be biased when there are large G-E and/or R-E correlations. Because of such correlations, the main genetic effects are over-adjusted when the phenotypes of the main trait are adjusted for the environmental variable in the model. Therefore, a careful interpretation of the estimated main genetic effects is required when using GxEsum. Fourth, we did not investigate the performance of GxEsum for ascertained case-control studies in which cases are over-sampled. A further study is required to extend the method to non-random case-control samples so that it can be applied to consortium data with multiple case-control studies. Lastly, when using the same sample size, the precision of GxEsum is not better than GREML-based GxE methods, implying that the former is only useful when using a large sample size that the latter cannot handle.

## Conclusions

Despite these caveats, GxEsum can be a useful tool to estimate whole-genome GxE as it can achieve a higher precision (i.e., power) from a larger sample size, compared to existing GxE methods. Especially when the scale of available resources increases, GxEsum may be a unique method that can be applied to large-scale data across multiple complex traits and diseases in the context of GxE.

## Methods

### GxEsum

Following Ni et al. [9], RNM can be written as:

$$ \mathbf{y}=\mathbf{b}+\mathbf{g}+\boldsymbol{\uptau} =\mathbf{b}+{\mathbf{g}}_{\mathbf{0}}+{\mathbf{g}}_{\mathbf{1}}\times \mathbf{E}+{\boldsymbol{\uptau}}_{\mathbf{0}}+{\boldsymbol{\uptau}}_{\mathbf{1}}\times \mathbf{E} $$

where **y** is the *N* vector of phenotypic observations; **b** is a vector of fixed effects; **g** is the *N* individual genetic effects, which can be decomposed into the first and second order of genetic random regression coefficients, **g**_**0**_ and **g**_**1**_; **τ** is the residual effects, decomposed into the first and second order of residual random regression coefficients, **τ**_**0**_ and **τ**_**1**_; and **E** is the *N* vector of environmental variable. Note that **E** can be also any covariate variable (e.g., smoking, alcohol intake frequency).

Assuming that the phenotypes (**y**) are pre-adjusted for the main genetic effects (**g**_**0**_), environmental or covariate variable (**E**), and other fixed effects (**b**), the model can be rewritten as:

$$ \mathbf{y}={\mathbf{g}}_{\mathbf{1}}\times \mathbf{E}+{\boldsymbol{\uptau}}_{\mathbf{0}}+{\boldsymbol{\uptau}}_{\mathbf{1}}\times \mathbf{E}=\mathbf{X}{\boldsymbol{\upbeta}}_{\mathbf{1}}\times \mathbf{E}+{\boldsymbol{\uptau}}_{\mathbf{0}}+{\boldsymbol{\uptau}}_{\mathbf{1}}\times \mathbf{E} $$

where **X** is an *N* x *M* standardized genotype matrix for *M* SNPs, and **β**_**1**_ is an *M* vector of SNP interaction effects modulated by the environment (i.e., GxE SNP effects). It is noted that **τ**_**0**_ is the residual effects that are consistent across the environment whereas **τ**_**1**_ captures the heterogeneous residual effects across the environment (i.e., RxE).

Following Bulik-Sullivan et al. [10], assuming $\mathbbm{E}\left[{\mathbf{g}}_{\mathbf{1}}\right]=\mathbbm{E}\left[{\boldsymbol{\upbeta}}_{\mathbf{1}}\right]=0$, the expected chi-square statistics of variant *j* for the GxE is:

1 $$ \mathbbm{E}\left[{\chi}_j^2\right]=N\bullet \mathrm{Var}\Big(\hat{\beta_{1j}\Big)} $$

Using the law of total variance, $\mathrm{Var}\Big[\hat{\beta_{1j}\Big]}$ can be obtained as:

$$ \mathrm{Var}\Big(\hat{\beta_{1j}\Big)}=\mathbbm{E}\left[\mathrm{Var}\left(\hat{\beta_{1j}}|\mathbf{EX}\right)\right]+\mathrm{Var}\left[\mathbbm{E}\left(\hat{\beta_{1j}}|\mathbf{EX}\right)\right] $$

$$ =\mathbbm{E}\left[\mathrm{Var}\left(\hat{\beta_{1j}}|\mathbf{EX}\right)\right] $$

where **EX** is an *N* x *M* matrix with each column having the Hadamard product between **E** and **X**_*j*_ (standardized genotypes at the *j*th SNP), and the conditional expectation of $\hat{\beta_{1j}}$ is $\mathbbm{E}\left(\hat{\beta_{1j}}|\mathbf{EX}\right)=0$.

Noting that the least-square estimate of $\hat{\beta_{1j}}$ can be obtained as $\hat{\beta_{1j}}=\left(\mathbf{E}{\mathbf{X}}_j\right)^{\prime}\mathbf{y}/N$, $\mathrm{Var}\left(\hat{\beta_{1j}}|\mathbf{EX}\right)$ can be rearranged as:

$$ \mathrm{VAR}\left(\hat{\beta_{1j}}|\mathbf{EX}\right)=\mathrm{Var}\left[\left(\mathbf{E}{\mathbf{X}}_j\right)^{\prime}\mathbf{y}/N|\mathbf{EX}\right] $$

$$ =\frac{1}{N^2}\mathrm{Var}\left[{\left(\mathbf{E}{\mathbf{X}}_j\right)}^{\prime}\mathbf{y}|\mathbf{EX}\right] $$

$$ =\frac{1}{N^2}{\left(\mathbf{E}{\mathbf{X}}_j\right)}^{\prime}\mathrm{Var}\left(\mathbf{y}|\mathbf{EX}\right)\left(\mathbf{E}{\mathbf{X}}_j\right) $$

$$ =\frac{1}{N^2}\left(\mathbf{E}{\mathbf{X}}_j\right)^{\prime}\mathrm{Var}\left[\left(\mathbf{E}\mathbf{X}\right){\boldsymbol{\upbeta}}_{\mathbf{1}}+{\boldsymbol{\uptau}}_{\mathbf{0}}+\mathbf{E}{\boldsymbol{\uptau}}_{\mathbf{1}}|\mathbf{EX}\right]\left(\mathbf{E}{\mathbf{X}}_j\right) $$

$$ =\frac{1}{N^2}{\left(\mathbf{E}{\mathbf{X}}_j\right)}^{\prime}\mathrm{Var}\left[\left(\mathbf{E}\mathbf{X}\right){\boldsymbol{\upbeta}}_{\mathbf{1}}|\mathbf{EX}\right]\left(\mathbf{E}{\mathbf{X}}_j\right) $$

$$ +\frac{1}{N^2}{\left(\mathbf{E}{\mathbf{X}}_j\right)}^{\prime}\left(\mathbf{E}{\mathbf{X}}_j\right)\mathrm{Var}\left({\boldsymbol{\uptau}}_{\mathbf{0}}\right)+\frac{1}{N^2}\left(\mathbf{E}{\mathbf{X}}_j\right)^{\prime}\left(\mathbf{E}\right)\left(\mathbf{E}\right)^{\prime}\left(\mathbf{E}{\mathbf{X}}_j\right)\mathrm{Var}\left({\boldsymbol{\uptau}}_{\mathbf{1}}\right) $$

$$ =\frac{1}{N^2}\left(\mathbf{E}{\mathbf{X}}_j\right)^{\prime}\left(\mathbf{E}\mathbf{X}\right){\left(\mathbf{E}\mathbf{X}\right)}^{\prime}\left(\mathbf{E}{\mathbf{X}}_j\right)\mathrm{Var}\left({\boldsymbol{\upbeta}}_{\mathbf{1}}|\mathbf{EX}\right) $$

$$ +\frac{1}{N^2}\left[N\mathrm{Var}\left({\boldsymbol{\uptau}}_{\mathbf{0}}\right)+\left(\mathbf{E}{\mathbf{X}}_j\right)^{\prime}\left(\mathbf{E}\right)\left(\mathbf{E}\right)^{\prime}\left(\mathbf{E}{\mathbf{X}}_j\right)\mathrm{Var}\left({\boldsymbol{\uptau}}_{\mathbf{1}}\right)\right] $$

$$ =\frac{1}{N^2}\frac{h_{{\boldsymbol{g}}_{\mathbf{1}}}^{\mathbf{2}}}{M}{\left(\mathbf{E}{\mathbf{X}}_j\right)}^{\prime}\left(\mathbf{E}\mathbf{X}\right){\left(\mathbf{E}\mathbf{X}\right)}^{\prime}\left(\mathbf{E}{\mathbf{X}}_j\right) $$

$$ +\frac{1}{N^2}\left[N\left(1-{h}_{{\boldsymbol{g}}_{\mathbf{1}}}^{\mathbf{2}}-{h}_{{\boldsymbol{\tau}}_{\mathbf{1}}}^{\mathbf{2}}\right)+{h}_{{\boldsymbol{\tau}}_{\mathbf{1}}}^{\mathbf{2}}\left(\mathbf{E}{\mathbf{X}}_j\right)^{\prime}\left(\mathbf{E}\right)\left(\mathbf{E}\right)^{\prime}\left(\mathbf{E}{\mathbf{X}}_j\right)\right] $$

where ${h}_{{\boldsymbol{g}}_{\mathbf{1}}}^{\mathbf{2}}$ and ${h}_{{\boldsymbol{\tau}}_{\mathbf{1}}}^{\mathbf{2}}$ are the proportion of phenotypic variance explained by GxE and RxE, respectively.

Therefore, $\mathbbm{E}\left[{\chi}_j^2\right]$ in Eq. (1) can be written as:

$$ \mathbbm{E}\left[{\chi}_j^2\right]=N\bullet \mathrm{Var}\left(\hat{\beta_{1j}}|\mathbf{EX}\right) $$

2 $$ =\frac{1}{N}\left[\frac{h_{{\boldsymbol{g}}_{\mathbf{1}}}^{\mathbf{2}}}{M}{\left(\mathbf{E}{\mathbf{X}}_j\right)}^{\prime}\left(\mathbf{E}\mathbf{X}\right){\left(\mathbf{E}\mathbf{X}\right)}^{\prime}\left(\mathbf{E}{\mathbf{X}}_j\right)+N\left(1-{h}_{{\boldsymbol{g}}_{\mathbf{1}}}^{\mathbf{2}}-{h}_{{\boldsymbol{\tau}}_{\mathbf{1}}}^{\mathbf{2}}\right)+{h}_{{\boldsymbol{\tau}}_{\mathbf{1}}}^{\mathbf{2}}\left(\mathbf{E}{\mathbf{X}}_j\right)^{\prime}\left(\mathbf{E}\right)\left(\mathbf{E}\right)^{\prime}\left(\mathbf{E}{\mathbf{X}}_j\right)\right] $$

According to Bulik-Sullivan et al. [10], the products of the standardized genotypes at variant *j* and other variants can be expressed as a function of LD scores, i.e.:

$$ \frac{1}{N^2}{\left({\mathbf{X}}_{\mathbf{j}}\right)}^{\prime}\left(\mathbf{X}\right){\left(\mathbf{X}\right)}^{\prime}\left({\mathbf{X}}_{\mathbf{j}}\right)=\frac{1}{N^2}\left({\mathrm{N}}^2+\left[\mathrm{N}\ast \left(1-\tilde{r}_{j}^2\right)+\tilde{r}_{j}^2\ast {\mathrm{N}}^2\right]\ast \left(\mathrm{M}-1\right)\right) $$

$$ =\mathbf{1}+\left[\frac{1-\tilde{r}_{j}^2}{\mathrm{N}}+\tilde{r}_{j}^2\right]\ast \left(\mathrm{M}-1\right) $$

$$ ={\ell}_j+\left[\frac{\left(\mathrm{M}-1\right)\left(1-\tilde{r}_{j}^2\right)}{\mathrm{N}}\right] $$

$$ \approx {\ell}_j+\frac{M-{\ell}_j}{N} $$

where $\tilde{r}_{j}^2$ is defined as the expected sample correlation between genotypes at the *j*th variant and the other (*M*-1) variants, and *ℓ*_*j*_ ***=*** 1***+*** $\tilde{r}_{j}^2\left(M-1\right)$ is the LD scores of the *j*th SNP.

According to the central moment theory of standard normal distribution of three independent random variables (**X**_**1**_, **X**_**2**_ and **E**), each with an N vector, useful equations are:

$$ \mathbbm{E}\left[\left({\mathbf{X}}_{\mathbf{1}}\right)^{\prime}\left({\mathbf{X}}_{\mathbf{1}}\right)\right]=N $$

$$ \mathbbm{E}\left[\left({\mathbf{X}}_{\mathbf{1}}\right)^{\prime}\left({\mathbf{X}}_{\mathbf{2}}\right)\left({\mathbf{X}}_{\mathbf{2}}\right)^{\prime}\left({\mathbf{X}}_{\mathbf{1}}\right)\right]=N $$

$$ \mathbbm{E}\left[\left({\mathbf{X}}_{\mathbf{1}}\right)^{\prime}\left({\mathbf{X}}_{\mathbf{1}}\right)\left({\mathbf{X}}_{\mathbf{1}}\right)^{\prime}\left({\mathbf{X}}_{\mathbf{1}}\right)\right]={N}^2 $$

$$ \mathbbm{E}\left[\left(\mathbf{E}{\mathbf{X}}_{\mathbf{1}}\right)^{\prime}\left(\mathbf{E}\right)\left(\mathbf{E}\right)^{\prime}\left(\mathbf{E}{\mathbf{X}}_{\mathbf{1}}\right)\right]=\mathbbm{E}\left[\left(\mathbf{E}{\mathbf{X}}_{\mathbf{1}}\right)^{\prime}\left(\mathbf{E}{\mathbf{X}}_{\mathbf{2}}\right)\left(\mathbf{E}{\mathbf{X}}_{\mathbf{2}}\right)^{\prime}\left(\mathbf{E}{\mathbf{X}}_{\mathbf{1}}\right)\right]=3N $$

and

$$ \mathbbm{E}\left[\left(\mathbf{E}{\mathbf{X}}_{\mathbf{1}}\right)^{\prime}\left(\mathbf{E}{\mathbf{X}}_{\mathbf{1}}\right)\left(\mathbf{E}{\mathbf{X}}_{\mathbf{1}}\right)^{\prime}\left(\mathbf{E}{\mathbf{X}}_{\mathbf{1}}\right)\right]={N}^2. $$

Therefore, assuming that **E** and **X**_**j**_ have a negligible correlation for a polygenic trait (i.e., a tiny proportion of the phenotypic variance of E can be explained by a single SNP, X_*j*_), the term (**EX**_*j*_)^′^(**EX**)(**EX**)^′^(**EX**_*j*_) can be expressed as a function of LD scores as:

$$ \frac{1}{N^2}{\left(\mathbf{E}{\mathbf{X}}_j\right)}^{\prime}\left(\mathbf{E}\mathbf{X}\right){\left(\mathbf{E}\mathbf{X}\right)}^{\prime}\left(\mathbf{E}{\mathbf{X}}_j\right)=\frac{1}{N^2}\left({N}^2+\left[3N\left(1-\tilde{r}_{j}^2\right)+\tilde{r}_{j}^2\ast {N}^2\right]\ast \left(M-1\right)\right) $$

$$ =1+\left[\frac{3\left(1-\tilde{r}_{j}^2\right)}{N}+\tilde{r}_{j}^2\right]\ast \left(M-1\right) $$

$$ ={\ell}_j+\frac{3\left(M-{\ell}_j\right)}{N} $$

Thus, a part in Eq. (2) can be rearranged as:

$$ \frac{1}{N}\left[\frac{h_{{\boldsymbol{g}}_{\mathbf{1}}}^{\mathbf{2}}}{M}{\left(\mathbf{E}{\mathbf{X}}_j\right)}^{\prime}\left(\mathbf{E}\mathbf{X}\right){\left(\mathbf{E}\mathbf{X}\right)}^{\prime}\left(\mathbf{E}{\mathbf{X}}_j\right)+N\left(1-{h}_{{\boldsymbol{g}}_{\mathbf{1}}}^{\mathbf{2}}\right)\right] $$

$$ =\frac{1}{N}\left[\frac{N^2{h}_{{\boldsymbol{g}}_{\mathbf{1}}}^{\mathbf{2}}}{M}\left({\ell}_j+\frac{3\left(M-{\ell}_j\right)}{N}\right)+N\left(1-{h}_{{\boldsymbol{g}}_{\mathbf{1}}}^{\mathbf{2}}\right)\right] $$

$$ =\frac{N{h}_{{\boldsymbol{g}}_{\mathbf{1}}}^{\mathbf{2}}}{M}\left({\ell}_j+\frac{3\left(M-{\ell}_j\right)}{N}\right)+1-{h}_{{\boldsymbol{g}}_{\mathbf{1}}}^{\mathbf{2}} $$

$$ =\frac{\left(N-3\right){h}_{{\boldsymbol{g}}_{\mathbf{1}}}^{\mathbf{2}}}{M}{\ell}_j+1+2{h}_{{\boldsymbol{g}}_{\mathbf{1}}}^{\mathbf{2}} $$

$$ =\frac{N\left(1-3/N\right){h}_{{\boldsymbol{g}}_{\mathbf{1}}}^{\mathbf{2}}}{M}{\ell}_j+1+2{h}_{{\boldsymbol{g}}_{\mathbf{1}}}^{\mathbf{2}} $$

3 $$ =\frac{N{h}_{{\boldsymbol{g}}_{\mathbf{1}}}^{\mathbf{2}}}{M}{\ell}_j+1+2{h}_{{\boldsymbol{g}}_{\mathbf{1}}}^{\mathbf{2}} $$

The term, 1 − 3/*N*, in Eq. (3) can be approximated as 1 in the analysis using biobank scale data, which contains over 10^5^ samples.

The remaining part in Eq. (2) can be rearranged as:

$$ \frac{1}{N}\left[N\left(-{h}_{{\boldsymbol{\tau}}_{\mathbf{1}}}^{\mathbf{2}}\right)+{h}_{{\boldsymbol{\tau}}_{\mathbf{1}}}^{\mathbf{2}}\left(\mathbf{E}{\mathbf{X}}_j\right)^{\prime}\left(\mathbf{E}\right)\left(\mathbf{E}\right)^{\prime}\left(\mathbf{E}{\mathbf{X}}_j\right)\right]=2{h}_{{\boldsymbol{\tau}}_{\mathbf{1}}}^{\mathbf{2}} $$

where $\mathbbm{E}\left[\left(\mathbf{E}{\mathbf{X}}_{\mathbf{1}}\right)^{\prime}\left(\mathbf{E}\right)\left(\mathbf{E}\right)^{\prime}\left(\mathbf{E}{\mathbf{X}}_{\mathbf{1}}\right)\right]=3N$ according to the central moment theory of standard normal distribution (see above), assuming that **E** and each column of **X** have a negligible correlation, which satisfies if **E** is an environmental variable or a polygenic trait.

Therefore,

4 $$ \mathbbm{E}\left[{\chi}_j^2\right]=N\bullet \mathrm{Var}\left(\hat{\beta_{1j}}|\mathbf{EX}\right)=\frac{N{h}_{{\boldsymbol{g}}_{\mathbf{1}}}^{\mathbf{2}}}{M}{\ell}_j+1+2{h}_{{\boldsymbol{g}}_{\mathbf{1}}}^{\mathbf{2}}+2{h}_{{\boldsymbol{\tau}}_{\mathbf{1}}}^{\mathbf{2}} $$

where $1+2{h}_{{\boldsymbol{g}}_{\mathbf{1}}}^{\mathbf{2}}+2{h}_{{\boldsymbol{\tau}}_{\mathbf{1}}}^{\mathbf{2}}$ can be obtained as the intercept of the outcome by fitting to the proposed model (GxEsum). It is noted Eq. (4) is valid when **X**_*j*_ is not strictly normally distributed, i.e., the centered and standardized genotypes of *j*th SNP, which is already shown in Bulik-Sullivan et al. [10]. When the environmental variable (**E**) is non-normal, the general form of the fourth central moment term can be expressed as $\mathbbm{E}\left[\left(\mathbf{E}{\mathbf{X}}_{\mathbf{1}}\right)^{\prime}\left(\mathbf{E}\right)\left(\mathbf{E}\right)^{\prime}\left(\mathbf{E}{\mathbf{X}}_{\mathbf{1}}\right)\right]=$*k*_*urtosis*_∙*N* where *k*_*urtosis*_ is the kurtosis of **E**. And only the intercept part of the Eq. (4) is slightly modified as:

5 $$ \mathbbm{E}\left[{\chi}_j^2\right]=N\bullet \mathrm{Var}\left(\hat{\beta_{1j}}|\mathbf{EX}\right)=\frac{N{h}_{{\boldsymbol{g}}_{\mathbf{1}}}^{\mathbf{2}}}{M}{\ell}_j+1+\left({\mathrm{k}}_{\mathrm{urtosis}}-1\right){h}_{{\boldsymbol{g}}_{\mathbf{1}}}^{\mathbf{2}}+\left({\mathrm{k}}_{\mathrm{urtosis}}-1\right){h}_{{\boldsymbol{\tau}}_{\mathbf{1}}}^{\mathbf{2}} $$

Equations (4) and (5) are verified using simulations (see Additional file 1: Note S3 and Table S2).

To validate the proposed model in general, we used comprehensive phenotypic simulations that were based on real genotype data (see Additional file 1: Note S4).

### Real data

UK Biobank data were used, which contains 0.5 million individuals aged between 40 and 69 years. The data consists of health-related information for each participant who was recruited in 2006–2010, and their imputed genomic data (~ 92 million SNPs) has been distributed through European Genome-phenome Archive. A stringent quality control process for individuals was set as follows: (1) who were reported as non-white British, (2) who were having mismatched gender between the reported and the inferred by the genotypic data, (3) who were having missing rate over 0.05, and (4) who were having putative sex chromosome aneuploidy. In addition, only HapMap3 SNPs were used which were passed from the stringent quality controls for SNPs. The filter for SNPs is set as follows: (1) which were having INFO score less than 0.6, (2) which were having a MAF less than 1%, (3) which were having Hardy-Weinberg Equilibrium (HWE) P-value less than 1E−4, and (4) one of which from the duplicated SNPs. From those passing the tough procedures, we additionally excluded one of pair of samples who were having a genomic relationship higher than 0.05. After quality control, 288,837 individuals and 1,133,273 SNPs remained. We estimated LD scores using the genotypic data of UK Biobank after these quality control processes.

Among the trait phenotypes available in the UK biobank, we arbitrarily selected BMI (a quantitative trait) and hypertension and type 2 diabetes (binary disease traits) and tested if the genetic effects of the complex traits were significantly modulated by an environmental variable, i.e., NEU, ALC, PA, or age (for testing BMI); BMI, WHR, or BFP (for hypertension); and BMI, diastolic BP, or systolic BP (for type 2 diabetes). The number of cases for hypertension and type 2 diabetes was 134,499 (population prevalence is 0.51) and 11,694 (population prevalence is 0.04), respectively. The phenotypes of the main trait were adjusted for potential confounders such as age, gender, year of birth, assessment center, Townsend Deprivation Index, genetic batch, household income, educational qualification [38], the first 10 principal components, and the environmental variable. For any phenotypic missing value for each variable, we used the mean of the phenotypes of the variable, i.e., phenotypic imputation with the mean. A better phenotypic imputation method [27] can be used, which is likely to improve the significance of GxE. Further details of the variables used in this study are in Additional file 1: Note S4.

In GWAS, we used a linear model for quantitative traits as well as for binary responses. The use of a linear model applied to binary responses is because it has been reported that a logistic regression may generate biased estimates in some instances [39], and our simulations (Additional file 1: Note S2) were based on a probit model (i.e., a linear transformation of the inverse standard normal distribution) that can be well approximated by a linear model [40].

## Supplementary Information

**Additional file 1.** Supplementary materials. It includes all supplementary notes, figures and tables.

**Additional file 2.** Review history.

## Data Availability

Data: The UK Biobank data are accessed via https://www.ukbiobank.ac.uk/ [41]. The ARIC study data are accessed via dbGaP (https://dbgap.ncbi.nlm.nih.gov), and its accession code is phs000280.v7.p1 [42]. Software: GxEsum model is implemented in the script that is publicly available at https://github.com/honglee0707/GxEsum [43], and the demonstration of GxEsum software is described in Additional file 1: Note S5. The version of source code used in the manuscript is deposited with DOI: 10.5281/zenodo.4659681 at https://zenodo.org/record/4659681#.YGkZXc9xeUk. The source code is under GNU General Public License v3.0. LDSC can be downloaded from https://github.com/bulik/ldsc [10]. PLINK version 1.9 can be downloaded from https://www.cog-genomics.org/plink/1.9/ [44]. MTG2 [45] version 2.15 can be downloaded from https://sites.google.com/site/honglee0707/mtg2.
