# Supplementary material for: GxEsum: a novel approach to estimate the phenotypic variance explained by genome-wide GxE interaction based on GWAS summary statistics for biobank-scale data
Source: Genome Biol. 2021 Jun 21;22:183. doi: 10.1186/s13059-021-02403-1 (PMC8218431; doi:10.1186/s13059-021-02403-1)
Supplement: Supplementary file 2 — Additional file 2. Review history. [file 13059_2021_2403_MOESM2_ESM.docx]

Review History

**First round of review**

**Reviewer 1**

**Were you able to assess all statistics in the manuscript, including the appropriateness of statistical tests used?**

Yes: This paper describes a new statistical method for assessing gene-environment interactions across the whole genome using summary statistics. The statistical methodology is a novel extension of existing work (LD score as specified by the authors). I was able to understand the derivations outlined in the Methods section, and the theory described in the Supplementary Materials. Additionally, simulations were used to confirm the theory and to quantify the performance of the method for a variety of input values. The simulations were very well described in the Supplementary Materials, and the statistics used to assess the performance of GxEsum in these simulations were appropriate and clearly explained. Similarly for the statistics used in the real-world example.

**Were you able to directly test the methods?**

No

**Comments to author:**

This paper provides a novel summary statistics based method to test for, and estimate, whole genome gene-environment interactions, in the presence of genetic and residual correlations between the outcome and the covariate traits, and whilst adjusting for residual-environment interactions. It does this by extending LD score regression to incorporate interactions and can perform analyses at speed within large-scale datasets, such as biobanks.

The authors have run simulations which they use to discuss when this method is most appropriate, and acknowledge the times it performs less well, for example, when GxEsum may not provide unbiased variance-covariance estimates. Additionally, analyses for two real world outcome trait examples are performed; one quantitative and one binary.

Although parameter estimates from GxEsum may not always be unbiased, the speed of this method for very large sample sizes, coupled with the controlled type 1 error rate, makes it useful for whole genome gene-by-environment interaction hypothesis testing. This allows researchers to identify significant GxE relationships for future study. Even in presence of collider bias the type 1 error rate is controlled, making this method appealing for use with summary statistics from biobank datasets, where collider bias has been highlighted as a potential problem (e.g. UK biobank).

The paper is a valuable addition to the literature, filling a gap in the toolkit for post GWAS analyses, and allowing researchers to explore the role environmental risk factors have in genetic studies. The novel GxE method is well-developed, carefully tested and should be widely used.

Minor comments

Comment 1

It would be useful to add a sentence into the Methods Overview section explicitly stating which summary statistics in practice are used to estimate σ^{2}_{g1} and σ^{2}_{τ1}. Here is what I believe to be the case:

'The chisq_{j} test statistic corresponds to β_{1j}, which is the effect size for the interaction between the j-th SNP (X_{j}) and the environmental covariate (E). The regression model from which the estimate for this is taken needs to be adjusted for the main effects (X_{j} and E) and other confounders if available. A regression model needs to be repeated for each SNP in turn.'

Although I can see in the Supplementary Material that you pre-adjust for confounders and the main effect of E, in which case the above would change to:

'The chisq_{j} test statistic corresponds to β_{1j}, which is the effect size for the interaction between the j-th SNP (X_{j}) and the environmental covariate (E). The outcome trait is pre-adjusted for confounders and the main effect of E, then a regression model with main effect X_{j} and the interaction (X_{j}xE) is run SNP-by-SNP'.

A sentence summarising this (or the actual case if the above is incorrect) would help readers understand the maths, and help them be clear what summary statistics they need to obtain so that they can use this method.

Comment 2

Either in the Methods section, or perhaps in the Supplementary Materials, a brief description for estimating σ^{2}_{g0} would be useful given that g0 is discussed in the simulations and the real data analysis. I suppose it follows from the LDSC methodology, but it should be made clear that the summary statistics now being used are those from the regression with the interaction term also included.

Comment 3

Pointing the reader towards the Supplementary Materials, where the simulation protocols are described in detail should be added into the Methods Overview section, paragraph 2. For example, 'For a full description of the simulation study please see the Supplementary Materials, section XXX'.

Comment 4

In the Simulation section, although it becomes clear in paragraph 4 when you state that the results that follow are for a binary trait, it would help the reader if you state that the results in the earlier paragraphs are for a continuous trait.

Comment 5

In the Simulation section (paragraph 4, sentence 3), you say:

'As shown in Supplementary Figure 5, the transformed estimates coincided the true simulated values on the liability scale although the precision of the estimates…'.

I assume that the 'transformed estimates coincided with the true simulated values' because the 95% CI for the variance estimate overlaps with the true GxE value. However, looking at Supplementary Figure 5 this appears questionable for some scenarios and prevalence values (the full scenario with k=0.5 and k=0.7). A comment to address this would be helpful- although I appreciate the likelihood of working with a disease with such prevalences may not be high.

Comment 6

In the Discussion you say you only use a first order random regression coefficients for the GxE interaction, and that additional higher-order interactions may be significant. If higher order interactions were present, would you expect the first order to be significant? That is, can GxEsum act as a filter to identify environmental covariates of interest that could then be further explored using reaction norm models, including checking for higher order interactions? And are there any clues to look for in the data that would suggest the presence of higher-order interactions?

Comment 7

Only one environmental variable can be used in GxEsum. Do you foresee extensions that would allow multiple variables? Or would a summary variable of the exposome used within the current GxEsum framework be the way to achieve this?

Possible typos

Section = background. Paragraph = 1. Sentence = 2.

Sentence reads: 'GWAS have been incredibly successful to identify genome-wide significant single nucleotide polymorphisms (SNPs) that are'…

Should 'to identify' be 'at identifying' instead?

Section = background. Paragraph = 4. Sentence = 2.

Sentence reads: 'In simulated and real data analyses, we show that the computational efficiency of the proposed approach is substantially higher than RNM, an existing GREML-based method while the estimates are reasonably accurate and precise.'

Missing comma between 'GREML-based method' and 'while the estimates…'?

Section = Results. Methods overview. Paragraph = 4 (last paragraph). Sentence = 1.

Sentence reads: 'Finally, we show how the genetic effects of trait (e.g. BMI or hypertension) are modulated by environment …'

Missing word? Could be something like:

Finally, we show how the genetic effects of a complex trait (e.g. BMI or hypertension) are …

Section = Simulations. Paragraph = 4. Sentence = 3.

Sentence reads: 'As shown in Supplementary Figure 5, the transformed estimates coincided the true simulated values on the liability scale although the precision of the estimates…'

Missing word, missing comma? Could be:

As shown in Supplementary Figure 5, the transformed estimates coincided with the true simulated values on the liability scale, although the precision of the estimates…'

Section = Simulations. Paragraph = 4. Sentence = 6.

Sentence reads: 'Note that the large GxE (> 5% of phenotypic variance) may not be realistic as estimated GxE explain only a small proportion…'

explain missing an s (explains) in above.

Section = Simulations. Paragraph = 5. Sentence = 2.

Sentence reads: 'We additionally tested if the type 1 error rate of GxEsum was controlled when there is collider bias, which is concerned especially when..'

Should 'concerned' be 'a concern'?

Section = Simulations. Paragraph = 5. Sentence = 3.

Sentence reads: 'In simulation with collider bias..'

Should 'simulation' be 'simulations'?

Section = Methods. End of page 17 in full document sent to reviewers. Below equation (4).

Reader is pointed to Supplementary table 11 to check intercept results- should this be table 3?

(Similarly in Supplementary Note 1, readers are pointed to table 8 but should perhaps be 3 again).

**Reviewer 2**

**Were you able to assess all statistics in the manuscript, including the appropriateness of statistical tests used?**

Yes: I was able to assess all statistical aspects of the m/s.

**Were you able to directly test the methods?**

Yes

**Comments to author:**

Shin et al present a novel approach (GxEsum) to estimate the genome-wide contribution of SNP-environment interactions to complex traits. Its major novelty that it relies on summary statistics from SNP-by-SNP GxE model. Through extensive simulation studies the authors show that their estimates are mostly unbiased and type I error is well-controlled. Finally, they apply the proposed method the BMI and hypertension as outcome with classical environments such as age, smoking, physical activity, alcohol use, etc. These analyses show a very modest contribution of GxE for these traits.

The method is solid and the derivations seem correct and elegant. The application is rather minimalistic (using the same variables as in previous papers by the authors), but since it is a primarily a methods paper, it is acceptable.

Major comments:

1. The authors rightly put that

E[(EX1)'(E)(E)'(EX1)] = E[(EX1)'(EX2)(EX2)'(EX1)] = 3N

However, if E is non-Gaussian with zero skewness, it becomes kurtosis*N. Does it lead to a final formula where all coefficients of "2" is replaced by "(kurtosis of E) - 1"? Also, adding skewness further impacts this number. How could a GxE heritability interpreted if it changed upon changing the scale of E (i.e. transforming)? In addition, the authors apply this to binary environment, where this does not hold at all and the multiplier depends on the case-control ratio.

2. The authors use several times in their derivations that G and E are uncorrelated, but have they tested in simulations that assuming a polygenic architecture for E with modest heritability (e.g. for smoking ~10% h2) does not lead to biased estimated?

3. I am somewhat puzzled to see that the GxE contribution seems to be very small here compared to what is found by other methods, such as LEMMA [https://www.biorxiv.org/content/10.1101/797829v2] or GPLEMMA [https://academic.oup.com/bioinformatics/advance-article/doi/10.1093/bioinformatics/btaa1079/6050717] or in comparison with Robinson et al. [https://pubmed.ncbi.nlm.nih.gov/28692066/] where GxSMK contributes 4% to BMI heritability, while here it is 0.5%. What are the reasons for these differences?

4. The BMI~GxSMK may not be the best example since there is a very clear BMI->SMK causal effect (positive), hence fitting such a model is not well-justified.

5. The code is very poorly commented and not really user-friendly to use. I understand that it requires simply to run a GxE GWAS, then subject the sum stats to LDSC, but it could be better wrapped around. The authors do not even provide the GxE estimates, it is left for the authors to compute it from the LDSC slope.

6. A limitation of the method is that if there are several E variables (the authors mention omics data), individual contributions can be established, but their overlap (between E1xG and E2xG) is not established and their multivariate interaction effect remains unknown (which could be a topic of future research e.g. what if Y ~ GXE1 + GxE2 + … GxEk model coefficients are known for each SNP?).

Minor comments:

1. It would be beneficial for the readers if at least some of the simulation results (concerning type I error control and bias) were included as a display item in the main manuscript.

2. The authors develop the theory assuming that X1, X2 and E are Gaussians, but in reality, X1 and X2 are (centered) binomials. I assume it does not cause any problem, but it would be handy to comment about the discrepancy.

**Reviewer 3**

**Were you able to assess all statistics in the manuscript, including the appropriateness of statistical tests used?**

Yes: I have some specific comments on the statistical tests in my comments to the authors

**Were you able to directly test the methods?**

No

**Comments to author:**

General Comments: Shin and Lee developed a summary statistics-based approach to estimating phenotypic variance explained by genome-wide gene-by-environment interactions (GxE). The analytical framework appears to be an extension of LD score regression for summary statistics-based estimation of SNP heritability (Bulik-Sullivan et al, 2015). While the authors showed that the proposed method had a clear advantage in computational efficiency compared with existing methods that use individual-level genetic data, I found that the manuscript is overall not well written and hard to follow. I have the following specific comments.

1. The title, abstract and introduction are not clear: the title "genotype-by-environment interaction model" "model" is too generic and vague, and "a method for estimating GxE" in the abstract is not clear about what GxE is being estimated. I was not sure about the objective of the manuscript until very late in the Introduction that the authors aimed to estimate the phenotypic variance explained by genome-wide GxE based on summary statistics.

2. The authors mixed use of type I error (hypothesis testing) and bias/standard error (point estimation). It should be stated clearly as to the null and alternative hypotheses in the current context, what test was used (I assume it was the Wald test). In addition, precision is different from the statistical power (the first sentence under "Precision and Computational Efficiency"). The authors need to make more clear connections between precision and power (e.g., under the Wald test with an unbiased/consistent point estimator).

3. It's not clear what types of summary statistics are needed: e.g., beta coefficients and their SEs for genetic main, E main and GxE terms? How about the LD between SNPs?

4. Demonstration of the accompanied software should be provided in the supplemental materials, including sample inputs, commands, outputs, and annotation to the outputs.

5. Can the proposed method be applied to consortia, e.g., case-control study consortium, with a large number of studies? What types of inputs would be needed, e.g., meta-analyzed beta's and SE? Any requirement on the scale and measure of the environmental variable across studies? This is an important issue since even large biobanks may not have enough cases for rare diseases to afford GxE analysis. Therefore, GxE analysis of rare/uncommon disease in the large-scale consortium context is more realistic.

6. The following statement is not supported by the presented analysis "We show that the causal relationship between hypertension and BMI is significantly explained by genome-wide GxE" (second paragraph in Discussion). I'm not convinced that GxE variance component analysis can have any implication on causal inference. Also it contradicts to the following statement "…like RNM, GxEsum does not determine the causal direction between variables" (second to last paragraph in Discussion).

7. The real data analysis. (a) the number of hypertension cases should be reported in the main text; (b) although it's a common practice to impute missing covariates, it's unusual to impute the missing phenotype values and imputation by mean seems over-simplified, which may have undue influence on the phenotypic variance estimation and partition; (c) the chosen phenotypes (BMI and hypertension) appeared to have much smaller GxE variance components that those of genetic main effects. Would this still hold for other traits/diseases? How about traits/diseases that are reported to have large GxE influence from family/pedigree studies? (d) Table 1: were the numbers in the "Main genetic effect" column from GCTA/LDscore or GEsum? Please report both to see if there was diluted main effects due to inclusion of GxE (over-adjustment as observed in the simulation study)

8. (minor) please provide a reference for RNM (second paragraph in Background)

9. (minor) Second paragraph in Results-Simulations "estimated GxE (g1) was unbiased": the mean of the estimated g1 across replications was numerically not equal to the true value, so it does not support "unbiasedness" exactly.

**Authors Response**

**Point-by-point responses to the reviewers’ comments:**

Reviewer #1: This paper provides a novel summary statistics based method to test for, and estimate, whole genome gene-environment interactions, in the presence of genetic and residual correlations between the outcome and the covariate traits, and whilst adjusting for residual-environment interactions. It does this by extending LD score regression to incorporate interactions and can perform analyses at speed within large-scale datasets, such as biobanks.

The authors have run simulations which they use to discuss when this method is most appropriate, and acknowledge the times it performs less well, for example, when GxEsum may not provide unbiased variance-covariance estimates. Additionally, analyses for two real world outcome trait examples are performed; one quantitative and one binary.

Although parameter estimates from GxEsum may not always be unbiased, the speed of this method for very large sample sizes, coupled with the controlled type 1 error rate, makes it useful for whole genome gene-by-environment interaction hypothesis testing. This allows researchers to identify significant GxE relationships for future study. Even in presence of collider bias the type 1 error rate is controlled, making this method appealing for use with summary statistics from biobank datasets, where collider bias has been highlighted as a potential problem (e.g. UK biobank).

The paper is a valuable addition to the literature, filling a gap in the toolkit for post GWAS analyses, and allowing researchers to explore the role environmental risk factors have in genetic studies. The novel GxE method is well-developed, carefully tested and should be widely used.

*Response: We thank the reviewer for this insightful summary including key messages of our paper.*

Minor comments

Comment 1

It would be useful to add a sentence into the Methods Overview section explicitly stating which summary statistics in practice are used to estimate σ^{2}_{g1}2 and σ^{2}_{τ1}. Here is what I believe to be the case:

'The chisq_{j} test statistic corresponds to β_{1j}, which is the effect size for the interaction between the j-th SNP (X_{j}) and the environmental covariate (E). The regression model from which the estimate for this is taken needs to be adjusted for the main effects (X_{j} and E) and other confounders if available. A regression model needs to be repeated for each SNP in turn.'

Although I can see in the Supplementary Material that you pre-adjust for confounders and the main effect of E, in which case the above would change to:

'The chisq_{j} test statistic corresponds to β_{1j}, which is the effect size for the interaction between the j-th SNP (X_{j}) and the environmental covariate (E). The outcome trait is pre-adjusted for confounders and the main effect of E, then a regression model with main effect X_{j} and the interaction (X_{j}xE) is run SNP-by-SNP'.

A sentence summarising this (or the actual case if the above is incorrect) would help readers understand the maths, and help them be clear what summary statistics they need to obtain so that they can use this method.

*R: We thank the reviewer for this helpful comment. We added ‘The χ_j^2 test statistics corresponds to the regression coefficient for the interaction between the jth SNP and the environmental covariate (E). The outcome trait is pre-adjusted for confounders and the main effect of E, then a regression model with the main and interaction effects is run by SNP-by-SNP' (line 15-18 in page 4)*

Comment 2

Either in the Methods section, or perhaps in the Supplementary Materials, a brief description for estimating σ^{2}{g0} would be useful given that g0 is discussed in the simulations and the real data analysis. I suppose it follows from the LDSC methodology, but it should be made clear that the summary statistics now being used are those from the regression with the interaction term also included.

*R: We added a description for estimating σ_(g_0)^2 (main genetic variance), which follows the LDSC methodology, in Supplementary Note 1, which is referred in the main text (line 6-8 in page 4).*

Comment 3

Pointing the reader towards the Supplementary Materials, where the simulation protocols are described in detail should be added into the Methods Overview section, paragraph 2. For example, 'For a full description of the simulation study please see the Supplementary Materials, section XXX'.

*R: We added “see Supplementary Note 2 for a full description of the simulation models”. (line 24 in page 4).*

Comment 4

In the Simulation section, although it becomes clear in paragraph 4 when you state that the results that follow are for a binary trait, it would help the reader if you state that the results in the earlier paragraphs are for a continuous trait.

*R: We added “For a continuous trait,” in the first paragraph of the Simulation section. (line 2 in page 5)*

Comment 5

In the Simulation section (paragraph 4, sentence 3), you say:

'As shown in Supplementary Figure 5, the transformed estimates coincided the true simulated values on the liability scale although the precision of the estimates…'.

I assume that the 'transformed estimates coincided with the true simulated values' because the 95% CI for the variance estimate overlaps with the true GxE value. However, looking at Supplementary Figure 5 this appears questionable for some scenarios and prevalence values (the full scenario with k=0.5 and k=0.7). A comment to address this would be helpful- although I appreciate the likelihood of working with a disease with such prevalences may not be high.

*R: We thank the reviewer for this comment. We estimated the variance component of GxE on the observed scale and transformed to that on the liability scale, using Robertson transformation. The transformation was originally derived for additive genetic variance (as shown in references #17 in the main text), which seems approximately valid for GxE variance (i.e. non-additive effects). In the main text, we explicitly discussed this with a caution (line 33 in page13 – line 3 in page 14). We also revised the sentence as “the transformed estimates were close to the true simulated values on the liability scale” (line 21 in page 6). In addition, we removed the simulation results with k=0.7 and 0.9 as such scenarios are unrealistic, to avoid any confusion (Supplementary Figures 5 and 12).*

Comment 6

In the Discussion you say you only use a first order random regression coefficients for the GxE interaction, and that additional higher-order interactions may be significant. If higher order interactions were present, would you expect the first order to be significant? That is, can GxEsum act as a filter to identify environmental covariates of interest that could then be further explored using reaction norm models, including checking for higher order interactions? And are there any clues to look for in the data that would suggest the presence of higher-order interactions?

*R: We agree that GxEsum can act as a filter to identify environmental covariates of interest that could then be further explored using reaction norm models, including checking for higher order interactions. It would be also possible to fit quadratic and polynomial functions of environmental covariates in GxEsum (i.e. the model can be written as y = g0 + g1xE + g2xE^2 + g3xE^3+, …, + residuals), which is, however, beyond the study scope. A further study is required for fitting higher order interactions and multiple environmental variables, using GWAS summary stats (discussed in line 10-13 in page 14).*

Comment 7

Only one environmental variable can be used in GxEsum. Do you foresee extensions that would allow multiple variables? Or would a summary variable of the exposome used within the current GxEsum framework be the way to achieve this?

*R: It can be naturally extended to fit multiple environmental variables (i.e. multiple regression that can be written as y = g0+ g1xE1 + g2xE2 +, …, + residuals). It is also an interesting suggestion to use a summary variable of the exposome in the GxEsum framework. However, these advanced models are not within the scope of this study. A further study is warranted for which we discussed in line 12 in page14 “…, assessing the performance of these advanced models is a formidable task, requiring a further study”*

Possible typos

Section = background. Paragraph = 1. Sentence = 2.

Sentence reads: 'GWAS have been incredibly successful to identify genome-wide significant single nucleotide polymorphisms (SNPs) that are'…

Should 'to identify' be 'at identifying' instead?

*R: We thank the reviewer for pointing out these typos. Corrected (line 5 in page 3)*

Section = background. Paragraph = 4. Sentence = 2.

Sentence reads: 'In simulated and real data analyses, we show that the computational efficiency of the proposed approach is substantially higher than RNM, an existing GREML-based method while the estimates are reasonably accurate and precise.'

Missing comma between 'GREML-based method' and 'while the estimates…'?

*R: Corrected (line 34 in page 3)*

Section = Results. Methods overview. Paragraph = 4 (last paragraph). Sentence = 1.

Sentence reads: 'Finally, we show how the genetic effects of trait (e.g. BMI or hypertension) are modulated by environment …'

Missing word? Could be something like:

Finally, we show how the genetic effects of a complex trait (e.g. BMI or hypertension) are …

*R: Revised (line 34 in page 4)*

Section = Simulations. Paragraph = 4. Sentence = 3.

Sentence reads: 'As shown in Supplementary Figure 5, the transformed estimates coincided the true simulated values on the liability scale although the precision of the estimates…'

Missing word, missing comma? Could be:

As shown in Supplementary Figure 5, the transformed estimates coincided with the true simulated values on the liability scale, although the precision of the estimates…'

*R: Revised (line 21 in page 6)*

Section = Simulations. Paragraph = 4. Sentence = 6.

Sentence reads: 'Note that the large GxE (> 5% of phenotypic variance) may not be realistic as estimated GxE explain only a small proportion…'

explain missing an s (explains) in above.

*R: Removed.*

Section = Simulations. Paragraph = 5. Sentence = 2.

Sentence reads: 'We additionally tested if the type 1 error rate of GxEsum was controlled when there is collider bias, which is concerned especially when..'

Should 'concerned' be 'a concern'?

*R: Corrected (line 7 in page 7)*

Section = Simulations. Paragraph = 5. Sentence = 3.

Sentence reads: 'In simulation with collider bias..'

Should 'simulation' be 'simulations'?

*R: Corrected (line 8 in page7)*

Section = Methods. End of page 17 in full document sent to reviewers. Below equation (4).

Reader is pointed to Supplementary table 11 to check intercept results- should this be table 3?

(Similarly in Supplementary Note 1, readers are pointed to table 8 but should perhaps be 3 again).

*R: Revised (lines 13-14 in page 18 and Supplementary Note 3)*

Reviewer #2: Shin et al present a novel approach (GxEsum) to estimate the genome-wide contribution of SNP-environment interactions to complex traits. Its major novelty that it relies on summary statistics from SNP-by-SNP GxE model. Through extensive simulation studies the authors show that their estimates are mostly unbiased and type I error is well-controlled. Finally, they apply the proposed method the BMI and hypertension as outcome with classical environments such as age, smoking, physical activity, alcohol use, etc. These analyses show a very modest contribution of GxE for these traits.

The method is solid and the derivations seem correct and elegant. The application is rather minimalistic (using the same variables as in previous papers by the authors), but since it is a primarily a methods paper, it is acceptable.

*R: We thank the reviewer for this positive comment.*

Major comments:

1. The authors rightly put that

E[(EX1)'(E)(E)'(EX1)] = E[(EX1)'(EX2)(EX2)'(EX1)] = 3N

However, if E is non-Gaussian with zero skewness, it becomes kurtosis*N. Does it lead to a final formula where all coefficients of "2" is replaced by "(kurtosis of E) - 1"? Also, adding skewness further impacts this number. How could a GxE heritability interpreted if it changed upon changing the scale of E (i.e. transforming)?

*R: We thank the reviewer for this insightful comment. When the environmental variable (E) is non-normal, the general form of the fourth central moment term can be expressed as E[(EX_1 )'(E)(E)'(EX_1 )]= kurtosis∙N where kurtosis is the kurtosis of E. And, only the intercept part of the Eq. (4) is slightly modified as*

*E[χ_j^2 ]=N∙Var((β_1j ) ̂|EX)=(Nh_(g_1)^2)/M l_j+1+(kurtosis-1)h_(g_1)^2+(kurtosis-1)h_(τ_1)^2.*

*This equation is now in Methods (eq. (5) in page 18).*

*Note that the derivation involves the fourth central moment, therefore, skewness (>0) would not affect the formular. Additional simulations were performed with a kurtosis of 6 and a skewness of 1 to verify Eq. (5) (Supplementary Table 2).*

In addition, the authors apply this to binary environment, where this does not hold at all and the multiplier depends on the case-control ratio.

*R: We applied GxEsum to age, neuroticism score, pack-years of smoking, physical activity, alcohol intake frequency, waist-hip ratio and body fat percentage that are not binary environment. We made this clearer in the legend of Table 3 and Supplementary Note 4 (Covariates section)). Nonetheless, we tested for binary E and found that the expectation from the theory (Eq. 5) and empirical observation agrees well (Supplementary Tables 2 and 3).*

2. The authors use several times in their derivations that G and E are uncorrelated, but have they tested in simulations that assuming a polygenic architecture for E with modest heritability (e.g. for smoking ~10% h2) does not lead to biased estimated?

*R: We clarified that the correlation between X_j and E are negligible because a tiny proportion of the phenotypic variance of E can be explained by X_j (i.e. a single SNP) for a polygenic trait (line 4-6 in page 17). In the simulation verification, we always simulated E with h2=0.5 (please see σ_γ^2 in Supplementary Note 2) and found that the assumption is not substantially biased (Supplementary Figures 1 - 4). We clarified this (i.e. simulated E with h2=0.5 ) in the legend of Supplementary Figures 1 - 4.*

3. I am somewhat puzzled to see that the GxE contribution seems to be very small here compared to what is found by other methods, such as LEMMA [https://www.biorxiv.org/content/10.1101/797829v2] or GPLEMMA [https://academic.oup.com/bioinformatics/advance-article/doi/10.1093/bioinformatics/btaa1079/6050717] or in comparison with Robinson et al. [https://pubmed.ncbi.nlm.nih.gov/28692066/] where GxSMK contributes 4% to BMI heritability, while here it is 0.5%. What are the reasons for these differences?

*R: We thank the reviewer for this comment. In LEMMA and GPLEMMA papers, ~ 7% of interaction variance is due to 42 environmental variables that include smoking status, physical activity, dietary and many other conditions (~ 0.2% for each environment on average, which approximately agrees with our results). In Robinson et al, as Ni et al. (2019) pointed out, their approach did not explicitly account for G-E correlation and residual heterogeneity when estimating GxE variance. Moreover, in their study, samples were arbitrarily stratified into four discrete groups. Owing to these factors, the result of Robinson et al. is not fully agreed with ours as well as those of LEMMA and GPLEMMA papers.*

4. The BMI~GxSMK may not be the best example since there is a very clear BMI->SMK causal effect (positive), hence fitting such a model is not well-justified.

*R: According to reviewer’s comment, we removed BMI~GxSMK result to avoid any confusion in this method article. Instead, we additionally analysed Type 2 diabetes ~ GxE and add the results in Table 3.*

5. The code is very poorly commented and not really user-friendly to use. I understand that it requires simply to run a GxE GWAS, then subject the sum stats to LDSC, but it could be better wrapped around. The authors do not even provide the GxE estimates, it is left for the authors to compute it from the LDSC slope.

*R: We improved the code, and it now provides the GxE estimates in its output. We also deposited the code in a GitHub and obtained a DOI via zenodo. All relevant information is now in Availability of Data and Materials section (line 19-23 in page 20). We also added Supplementary Note 5 (Demonstration of GxEsum software).*

6. A limitation of the method is that if there are several E variables (the authors mention omics data), individual contributions can be established, but their overlap (between E1xG and E2xG) is not established and their multivariate interaction effect remains unknown (which could be a topic of future research e.g. what if Y ~ GXE1 + GxE2 + … GxEk model coefficients are known for each SNP?).

*R: We thank the reviewer for this comment. It can be naturally extended to fit multiple environmental variables (i.e. multiple regression that can be written as y = g0+ g1xE1 + g2xE2 +, …, + gnxEn + residuals). We agree that investigating this advanced model can be a topic of future research. A further study is warranted for which we discussed in page 14 (line 10-13).*

Minor comments:

1. It would be beneficial for the readers if at least some of the simulation results (concerning type I error control and bias) were included as a display item in the main manuscript.

*R: We moved Supplementary Tables 1 and 4 into the main text (Tables 1 and 2).*

2. The authors develop the theory assuming that X1, X2 and E are Gaussians, but in reality, X1 and X2 are (centered) binomials. I assume it does not cause any problem, but it would be handy to comment about the discrepancy.

*R: We thank the reviewer for this comment. Eq. (4) is valid when X_j is not strictly normally distributed, i.e. the centred and standardised genotypes of jth SNP, which is already shown in Bulik-Sullivan et al. (ref. #10) and verified in our simulations. Furthermore, we tested non-normal E with substantial skewness and kurtosis and found that empirical observations agreed with expectations from theory (Supplementary Tables 2 and 3). We commented this in line 6–14 in page 18.*

Reviewer #3: General Comments: Shin and Lee developed a summary statistics-based approach to estimating phenotypic variance explained by genome-wide gene-by-environment interactions (GxE). The analytical framework appears to be an extension of LD score regression for summary statistics-based estimation of SNP heritability (Bulik-Sullivan et al, 2015). While the authors showed that the proposed method had a clear advantage in computational efficiency compared with existing methods that use individual-level genetic data, I found that the manuscript is overall not well written and hard to follow. I have the following specific comments.

*R: We would like to thank the reviewer for their helpful comments.*

1. The title, abstract and introduction are not clear: the title "genotype-by-environment interaction model" "model" is too generic and vague, and "a method for estimating GxE" in the abstract is not clear about what GxE is being estimated. I was not sure about the objective of the manuscript until very late in the Introduction that the authors aimed to estimate the phenotypic variance explained by genome-wide GxE based on summary statistics.

*R: According to the comment, we revised the Title as “GxEsum: a novel approach to estimate the phenotypic variance explained by genome-wide GxE interaction based on GWAS summary statistics for biobank-scale data”. In Abstract, we revised as “… GxEsum, a method for estimating the phenotypic variance explained by genome-wide GxE based on GWAS summary statistics” (line 6 in page 2). In Background, we added “… a novel approach to estimate the phenotypic variance explained by genome-wide GxE based on GWAS summary statistics” (line 29 in page 3).*

2. The authors mixed use of type I error (hypothesis testing) and bias/standard error (point estimation). It should be stated clearly as to the null and alternative hypotheses in the current context, what test was used (I assume it was the Wald test). In addition, precision is different from the statistical power (the first sentence under "Precision and Computational Efficiency"). The authors need to make more clear connections between precision and power (e.g., under the Wald test with an unbiased/consistent point estimator).

*R: We thank the reviewer for this comment. We removed ‘(i.e. power)’ in the first sentence in the section (line 21 in page 7), and made clearer by adding “GxEsum uses the Wald test to get a p-value for the null hypothesis, i.e. absence of GxE interaction, using an estimated GxE variance and its standard error. Therefore, the power of the method is closely related to the precision.” (line 1- 3 in page 8).*

3. It's not clear what types of summary statistics are needed: e.g., beta coefficients and their SEs for genetic main, E main and GxE terms? How about the LD between SNPs?

*R: We clarified this (line 13 - 20 in page 4) as “…, l_jis the LD score at the variant j that can be estimated from a reference panel. The χ_j^2 test statistics corresponds to the regression coefficient for the interaction between the jth SNP and the environmental covariate (E). The outcome trait is pre-adjusted for confounders and the main effect of E, then a regression model with the main and interaction effects is run by SNP-by-SNP. If chi-square statistics from GWAS are regressed on LD scores, …”.*

4. Demonstration of the accompanied software should be provided in the supplemental materials, including sample inputs, commands, outputs, and annotation to the outputs.

*R: We added a demonstration into Supplementary Note 5.*

5. Can the proposed method be applied to consortia, e.g., case-control study consortium, with a large number of studies? What types of inputs would be needed, e.g., meta-analyzed beta's and SE? Any requirement on the scale and measure of the environmental variable across studies? This is an important issue since even large biobanks may not have enough cases for rare diseases to afford GxE analysis. Therefore, GxE analysis of rare/uncommon disease in the large-scale consortium context is more realistic.

*R: We thank the reviewer for this insightful comment. This is a limitation because GxEsum is designed to use population-based biobank data (as in the revised title). We added a limitation in Discussion as “Fourth, we did not investigate the performance of GxEsum for ascertained case-control studies in which cases are over-sampled. A further study is required to extend the method to non-random case-control samples so that it can be applied to consortium data with multiple case-control studies.” (line 18 – 21 in page 14)*

6. The following statement is not supported by the presented analysis "We show that the causal relationship between hypertension and BMI is significantly explained by genome-wide GxE" (second paragraph in Discussion). I'm not convinced that GxE variance component analysis can have any implication on causal inference. Also it contradicts to the following statement "…like RNM, GxEsum does not determine the causal direction between variables" (second to last paragraph in Discussion).

*R: We revised as “The causal relationship between hypertension and BMI, and type 2 diabetes and BMI reported in the previous studies (reference #24, 27 and 28) may be partly due to the genome-wide GxE interaction effects”. (lines 24-25 in page 12)*

7. The real data analysis.

(a) the number of hypertension cases should be reported in the main text;

*R: Added (line 40 in page 18)*

(b) although it's a common practice to impute missing covariates, it's unusual to impute the missing phenotype values and imputation by mean seems over-simplified, which may have undue influence on the phenotypic variance estimation and partition;

*R: We thank the reviewer for this comment. We explicitly tested GxEsum with and without imputation and found that there was no dramatic difference (Supplementary Tables 10 - 12), which was mentioned in the main text (line 17 in page 10). In fact, the proportion of missing data for the main trait is not so high (0.3% for BMI and 7.9% for hypertension, 0.2% for type 2 diabetes) (clarified in the main text (line 19 -23 in page 10) and in the legends of Supplementary Tables 10 - 12). We also added a reference for a better phenotypic imputation method (ref #21) (line 23 in page 10 and line 7 in page 19).*

(c) the chosen phenotypes (BMI and hypertension) appeared to have much smaller GxE variance components that those of genetic main effects. Would this still hold for other traits/diseases? How about traits/diseases that are reported to have large GxE influence from family/pedigree studies?

*R: We thank the reviewer for this comment. The traits and variables used in this Method paper are limited examples, and there may be a large GxE variance for other traits/diseases. We additionally analyse type 2 diabetes with a number of covariates and found a large GxE variance (Table 3). Given this result, we revised the main text accordingly (e.g. line 43 in page 13). We did not consider family/pedigree data, which however can have rich information to detect genetic effects (reference #35). It is interesting to investigate how GxEsum performs for family/pedigree data, which however is beyond the scope of this study and requires a further study.*

(d) Table 1: were the numbers in the "Main genetic effect" column from GCTA/LDscore or GEsum? Please report both to see if there was diluted main effects due to inclusion of GxE (over-adjustment as observed in the simulation study)

*R: We reported both LDSC and GxEsum estimates for the main additive genetic variance in Supplementary Table 9 (also mentioned in the main text (line 14, page 10)). Biased estimates of the main additive genetic variance due to over-adjustment of the phenotypes were not clearly observed in the real data analysis.*

8. (minor) please provide a reference for RNM (second paragraph in Background)

*R: Added (line 18 in page 3).*

9. (minor) Second paragraph in Results-Simulations "estimated GxE (g1) was unbiased": the mean of the estimated g1 across replications was numerically not equal to the true value, so it does not support "unbiasedness" exactly.

*R: We revised as “estimated GxE (g1) was not remarkably different from the true values …” (line 19 in page 5).*

**Second round of review**

**Reviewer 1**

Thank you for responding to my comments and queries about your manuscript, describing your genome-wide genotype-by-environment interaction summary statistics method (GxEsum). After reading the revised manuscript, and the responses to my comments, I have no further questions about the method or results presented that would impact publication. I think the computational speed of GxEsum, combined with the controlled Type 1 error rate for quantitative and binary traits, and the ability to include all available participants at biobank scale, make it a useful addition to the GxE interaction identification toolkit. The simulations and explanations make it clear when bias is observed and when caution should be applied to interpretation of parameter estimates. Additionally, the practical/ software run through in the Supplementary Materials is very useful. The changes to the phenotypes presented as examples seem appropriate and convey the utility of the method. My only remaining comment is that careful proofreading is required because there are some some minor sentence structure issues and typos remaining that impact the readability of the paper.

**Reviewer 2**

I’d like to congratulate the authors on the meticulous revision. All my comments were adequately addressed.

I only have one question left upon looking at the new T2D ~ BMI x G effects. This seems to be enormous and completely different from everything seen for other trait pairs. It would be very important to discuss these results more: does T2D ~ WHRxG shows similar effects? What surprises me is that BMI and DBP have a correlation of 0.3, so an DBPxG random effect is expected to explain at least ~9% (0.3^2) of the interaction effect of 0.085, i.e. 0.008, but the DBPxG estimate was highly compatible with zero. What was the correlation between the estimates for sigma_g0 and sigma_g1 for the T2D ~ WHRxG example?

Also, the Hypertension ~ BMI x G analysis should give a similar qualitative answer as the SBP ~ BMI x G test. What does it give?

**Reviewer 3**

The authors have satisfactorily addressed all my comments and concerns. The revised manuscript has been substantially improved over the original submission.

**Authors Response**

**Point-by-point responses to the reviewers’ comments:**

*We are pleased that you would, in principle, accept our manuscript once the remaining concerns from the Reviewer #2 are properly addressed. Referee 2 asked that we should add a bit more discussion of a surprising result, and we revised the third paragraph in Discussion (page 10) as*

*“…. The GxE interaction variance for type 2 diabetes is substantially larger than that for hypertension when using BMI as environmental exposure. This observation agrees with Hyppönen et al. (2019) [25] reporting that BMI genetic risk score is more strongly associated with type 2 diabetes than hypertension. In contrast, there is no significant evidence of genome-wide GxE for hypertension-WHR, hypertension-BFP, type 2 diabetes-DBP or type 2 diabetes-SBP causal relationship that was observed in Mendelian randomization studies [29-31]. This is not totally unexpected because type 2 diabetes or hypertension was reported to be more significantly associated with BMI than other variables [25, 28, 29].”*

*We also have read the manuscript to check typo and inconsistency to increase the readability (reviewer #1).*
